# The Relationship Between Sugar-Sweetened Beverages, Takeaway Dietary Pattern, and Psychological and Behavioral Problems Among Children and Adolescents in China

**DOI:** 10.3389/fpsyt.2021.573168

**Published:** 2021-08-12

**Authors:** Yi Zhang, Xiaoyan Wu, Qianling Wang, Qiao Zong, Renjie Wang, Tingting Li, Shuman Tao, Fangbiao Tao

**Affiliations:** ^1^Department of Maternal, Child and Adolescent Health, School of Public Health, Anhui Medical University, Hefei, China; ^2^Ministry of Education Key Laboratory of Population Health Across Life Cycle, Hefei, China; ^3^National Health Commission Key Laboratory of Study on Abnormal Gametes and Reproductive Tract, Hefei, China; ^4^Department of Nephrology, The Second Hospital of Anhui Medical University, Hefei, China

**Keywords:** sugar-sweetened beverages, take-away dietary patterns, strengths and difficulties questionnaire, children, adolescents, psychological and behavioral problems

## Abstract

**Background and Aim:** The association of sugar-sweetened beverage (SSB) consumption and takeaway dietary pattern with psychological problems in Chinese children and adolescents has not been concretely reported. Our study aimed to investigate the association between SSB consumption, takeaway dietary pattern, and psychological and behavioral problems (PBPs).

**Methods:** Cluster sampling method has been adopted from April to May 2019 to conduct a questionnaire survey among 30,188 children and adolescents in grades 1 to 12 from 14 schools in six streets in Bao'an District of Shenzhen. This cross-sectional study investigated the association of consumption of SSBs and takeaway patterns with PBPs, and PBPs were measured by the Strengths and Difficulties Questionnaire (SDQ) in primary, junior, and senior high school students.

**Results:** A total of 33,801 primary, junior, and senior high school students (mean age = 12.44, SD = 3.47) ranging from 6 to 18 years old were recruited in this study using a health survey of children and adolescents in junior and senior high schools (grades 1–12), and 30,188 students with no missing data were finally analyzed (questionnaires with missing value >5% were excluded). The top three SSBs in the intake frequency were milk beverage drinks (not milk), vegetable protein drinks, and fruit and vegetable juice drinks. Adjusted for demographic factors, the higher the frequency of students consuming SSBs who have significantly higher PBPs, the higher the frequency of students with takeaway dietary patterns who also have significantly higher PBPs. More frequent intake of SSBs [odds ratio (OR) = 2.23, 95%CI = 2.0–2.47, *p* < 0.01] and higher takeaway dietary patterns (OR = 2.34, 95%CI = 1.81–3.03, *p* < 0.01) were associated with higher SDQ total difficulties scores. When low and medium consumption of SSB was compared, children and adolescents who have high SSB intake were more associated with total difficulties score (OR = 3.10, 95%CI = 2.67–3.59, *p* < 0.01), and when low and medium takeaway dietary patterns were compared, children and adolescents who have high takeaway dietary patterns were more associated with total difficulties score. The joint associations of SSBs and takeaway pattern with SDQ were stronger than the associations individually.

**Conclusions:** Students consuming higher SSBs and having takeaway dietary pattern are associated with increased levels of PBPs individually and interactively. These results may have implications for mental health prevention in adolescents.

## Introduction

Throughout the past few decades, sugar-sweetened beverages (SSBs) account for most of the growth in global sugar consumption (1–3). In a previous study, we found that SSBs and foods with high sugar contents may cause obesity and carious disease, may stimulate chronic diseases, may also activate hyperactivity disorders (4) and obesity-related type 2 diabetes mellitus, cancers, metabolic syndrome, and cardiovascular disease (5, 6). Additionally, SSBs also contain other additives that could cause children's behavioral problems and obesity (7–10). As we have known, common daily SSBs include carbonated and fizzy drinks, sweetened milk and milky tea drinks, sparkling water, energy drinks, sweetened orange fruit drinks, sports and energy drinks, vitamin-water drinks, and vegetable protein drink (10–13). The 2015 Dietary Guidelines for Americans recommends limiting total added sugar intake to <10% of daily calories (14). Therefore, a series of problems caused by SSBs should also be noted.

It is worth noting that the impacts of SSBs on mental health have attracted widespread interest from researchers. Previous evidence suggests that the increase in sugar drinks consumption is thought to be a predictor and a result of mental health problems (15). A cross-sectional study explored the association between soft drinks, and hyperactivity and behavioral problems in adolescents (16). And we also have found in Australia that there is a correlation between the consumption of sugar drink and passively acquired mental health problems among adolescents (17). Some others also indicated similar results such as sugar consumption have being linked to an increased risk of attention deficit/hyperactivity disorder (ADHD), depression, and anxiety symptoms (13, 16–18). These results almost suggest that SSBs may have adverse effects on the mental health susceptibility of adolescents (19). These beverages should be consumed with caution, not only because excessive sugar intake can lead to an imbalance in caloric intake that affects a balanced diet but also because it may be associated with mental health problems (20–22), especially among school-aged children.

Previous studies have also shown that the proportion of food spending on eating out of home in Chinese cities rose from 7.9% in 1992 to 21.2% in 2010 (23), which is consistent with a longitudinal study with data collected from the China Health and Nutrition Survey, which found that eating away from home became more common (24). The tempo of life is becoming faster, more and more people also will chose convenient lifestyles, and one of the most obvious characteristics of fast foods and takeaway foods is comfort, which means they save extra time greatly (25, 26). However, in one longitudinal study, researches have found the associations between fast food and physical health (27, 28). Even so, with the rapid development of the economy, more and more electrical products appear in front of us. Many people are ordering fast food takeout online via electronic products (e.g., automobile phone) and ordering food at home (takeaway, takeout, and fast food), which has become more and more popular in the recent decades. These all belonged to unhealthy diet patterns (UDPs). Fast-food consumption is significantly and positively associated with total energy, total fat, saturated fat, total carbohydrate, added sugars, SSBs, and non-beverage energy density. The consumption of fast food and takeaway food represents a public health problem and has been found to be associated with overweight and poor diet quality, as these fast-food and takeaway food menus often contain foods high in energy and fat/sugar (29, 30). More than that, some studies have found significant independent associations between the takeaway dietary patterns and sweet and fast foods and the prevalence of mental health (31–35). For adolescents, a review of nine cohort and three cross-sectional studies confirmed the association between unhealthy diets (including fast food and takeaways) and an increased risk of mental illness (36).

Previous researches exactly reported the relationships between SSBs and takeaway dietary patterns (25, 37, 38). Relative fast-food and takeaway environment exposure was positively associated with SSB intake (39, 40). Given the growing data on children with mental health problems, there is an urgent need to fully identify underlying dietary intake problems, which may not only exacerbate these problems but may also contribute to physical health problems later in life for these growing children. Despite this evidence, the effects of other common diet components, such as fats and sugar, on mental health are still unclear. Furthermore, to our knowledge, few previous studies have analyzed the association between takeaway patterns, SSBs, and psychological behavioral problems among children and adolescents, and few have been about the interactive effect of this association. Based on the results of the reviews mentioned above that fast-food consumption and carbonated soft drink consumption in children and adolescents tend to co-occur, we hypothesized that there might be interactive effects of takeaway patterns and SSBs in the psychological behavioral problems among children and adolescents. In addition, our study explored multiple types of sugary drinks. In this study, we used data of cross-sectional investigation from China to analyze the interactive model. Therefore, our study aimed to investigate the individual and interactive relationships between SSBs, takeaway dietary pattern, and psychological and behavioral problems (PBPs) among Chinese children and adolescents.

## Methods

### Study Designs

We took into account both the sampling method and the partnership. We first contacted the Centers for Disease Control and Prevention (CDC) in Bao'an District of Shenzhen, and the local CDC finally selected six streets according to the geographical distribution and the degree of school cooperation. Fourteen schools were selected randomly. A total of 33,801 primary, junior, and senior high school students (mean age = 12.44, SD = 3.47) ranging from 6 to 18 years old were recruited in this study using a health survey of children and adolescents in junior and senior high schools (grades 1–12). In each school, a cluster sampling method was used to extract three classes from each grade. The participants were then asked to complete a questionnaire. Written informed consent and questionnaires were obtained from the students (grades 5–12) or their parents (grades 1–4) (one for parents (grades 1–4) and one for self-reporting (grades 5–12), so the grade 1–4 children obtained their consent from their parents, and consent from grade 5–12 students was obtained directly from them). There were two types of questionnaires: one for parents (grades 1–4) and one for self-reporting (grades 5–12). The survey was conducted from April to May 2019. Due to an unwillingness to respond to the questionnaire, absence from school, high levels of missing data (a questionnaire with missing value >5%), or obviously fictitious responses, 3,613 (10.7%) participants were excluded from the study. Thus, the data from 30,188 participants (response rate: 89.3%) were analyzed. This sample is well-represented in the general population of China, as it also involves almost all children's and adolescents' age groups, including primary, junior, and senior high school samples. This study was approved by the Ethics Committee of Anhui Medical University.

### Measures

We conducted a comprehensive questionnaire to measure some variables, including SSB intake, fast-food/takeaway consumption assessment of psychological behavioral problems, and demographic variables. Participants filled out questionnaires in separate classrooms where privacy was guaranteed. During the investigation, two or three investigators in each room provided technical support. The assessment of psychological behavioral problems was perfumed through the Strengths and Difficulties Questionnaire (SDQ) content on the questionnaire. SDQ includes 25 items, including hyperactivity problems, emotional symptoms, conduct problems, peer problems, and prosocial problems (41). The SDQ is a short screening instrument that addresses the positive and negative behavioral attributes of children and adolescents; the SDQ is widely used to evaluate child developmental disabilities, and psychological and psychiatric conditions or disorders (42); and SDQ scales have been used as a tool in various studies for child mental health and validated for internal consistency (43). Children's responses can be categorized as normal, borderline, or abnormal for each of the subscales. Total difficulties of SDQ scores above the 90th percentile were classified as abnormal; the 80th to 90th percentiles were classified as “borderline,” and those below the 80th percentile were classified as “normal” (44). The internal consistency of the SDQ total score was good (Cronbach's alpha 0.762) in our manuscript.

The frequency of carbonated, soda, tea and milk beverage, fruit and vegetable juice drinks, coffee drinks and energy drinks, and vegetable protein drink consumption has used in the questionnaire to figure out the total scores (45). Low SSB intake was defined as the lowest quintile (≤20th percentile) of the total score of SSBs serving equivalents per week; medium SSB intake was defined as the second to fourth quintiles (>20th to <80th percentile); and high SSB intake was defined as the highest quintile (≥80th percentile) (46). In our study, takeaway dietary pattern mainly refers to the takeaway behavior. Students were asked by one question: “How many times do you eat takeaways each week? (37)”. The frequency answers were never, one to two times, three to four times, and more than five times. The frequency was categorized as never, low frequency, medium frequency, and high frequency.

### Statistical Analysis

The database was created by EpiData 3.0. Statistical analyses were performed with SPSS 23.0 and GraphPad Prism. Descriptive analyses were used to show demographic information of the sample. Pearson's chi-squared tests were performed to test the prevalence of PBPs. Additionally, the dose–response trend test was conducted in the one-way analysis of variance. Multivariable logistic regression was used to explore the independent and interactive associations of takeaways, SSBs, and PBPs. GraphPad Prism was used to draw the correlation graphs. Models controlled for age, gender, grade, residential areas, family economic level, and parents' educational level. Odds ratios (ORs) and their 95% confidence intervals (CIs) were calculated. The significance level was set at *p* < 0.05.

## Results

### Participants

We first arranged staff to input the questionnaires, and then data were imported into SPSS. Questionnaires with missing values >5% were deleted. Then we use multiple imputation to fill in data with missing values <5%. In total, 33,801 students consented to participate and have completed the questionnaire. Excluding participants whose data were incomplete and did not meet the criteria (excluding questionnaires with a missing value of >5%), the final study had 30,188 participants. The sample's mean age was 12.44 years (SD = 3.47). There were 13,291 (44.0%) girls and 16,897 (56.0%) boys. The primary participants were 44.5%. There were no association between gender, age, and grade. Females were more likely to have SSBs and takeaway consumption. The characteristics of the participants' demographics are shown in Table 1.

**Table 1:** General characteristics of the samples, *n* (%).

**Variables**	**Total**	**Male**	**Female**	**χ^**2**^**
**Age (years)**				
≤ 12	14,484 (48.0)	8,127 (48.1)	6,357 (47.8)	0.21
>12	15,704 (52.0)	8,770 (51.9)	6,934 (52.2)	
**Grade**				
Primary	13,420 (44.5)	7,544 (44.6)	5,876 (44.2)	0.58
Junior high	8,232 (27.3)	4,590 (27.2)	3,642 (27.4)	
Senior high	8,536 (28.3)	4,763 (28.2)	3,773 (28.4)	
**Academic record**				
Good	7,580 (25.1)	4,246 (25.1)	3,334 (25.1)	73.65^**^
Medium	17,573 (58.2)	9,567 (56.6)	8,006 (60.2)	
Bad	5,035 (16.7)	3,084 (18.3)	1,951 (14.7)	
**Residential areas**				
Country	5,585 (18.5)	3,254 (19.3)	2,331 (17.6)	16.88^**^
Town	2,754 (9.1)	1,490 (8.8)	1,264 (9.5)	
City	21,849 (72.4)	12,153 (71.9)	9,696 (72.9)	
**Father's education**				
Junior high or lower	9,461 (31.9)	5,574 (33.0)	4,067 (30.6)	22.79^**^
Senior high	11,387 (37.7)	6,339 (37.5)	5,048 (38.0)	
Junior college or above	9,160 (30.3)	4,984 (29.5)	4,176 (31.4)	
**Mother's education**				
Junior high or lower	12,203 (40.5)	6,992 (41.4)	5,211 (39.2)	14.70^**^
Senior high	11,184 (37.0)	6,170 (36.5)	5,014 (37.7)	
Junior college or above	6,801 (22.5)	3,735 (22.1)	3,066 (23.1)	
**Family economic level**				
Under moderate	4,113 (13.6)	2,596 (15.4)	1,517 (11.4)	100.22^**^
Moderate	20,395 (67.6)	11,144 (66.0)	9,251 (69.6)	
Over moderate	5,680 (18.8)	3,157 (18.7)	2,523 (19.0)	
**SSB frequency**				
High intake	6,482 (21.5)	4,290 (25.4)	2,192 (16.5)	358.88^**^
Medium intake	15,623 (51.8)	8,421 (49.8)	7,202 (54.2)	
Low intake	8,083 (26.8)	4,186 (24.8)	3,897 (29.3)	
**Takeaway frequency**				
Never	17,183 (56.9)	9,788 (57.9)	7,395 (55.6)	26.86^**^
Low frequency	11,426 (37.8)	6,216 (36.8)	5,210 (39.2)	
Medium frequency	1,126 (3.7)	611 (3.6)	515 (3.9)	
High frequency	453 (1.5)	282 (1.7)	171 (1.3)	

*SSB, sugar-sweetened beverage*.

*p < 0.05;

** *p < 0.01*.

### Sugar-Sweetened Beverages, Takeaway, and Psychological and Behavioral Problem Symptoms (Strengths and Difficulties Questionnaire Scores)

The top three SSBs in the intake frequency were milk beverages drinks (not milk), vegetable protein drinks, and fruit and vegetable juice drinks. Intake of milk beverages drinks accounted for the largest proportion, while the intake frequency of vegetable protein drinks was more than twice as much as fruit and vegetable juice drinks across the overall intake frequency groups; soda drinks and energy drinks were the least frequent. In our results, 56.9, 37.8, 3.7, and 1.5% of adolescents' takeaway dietary pattern was never, high frequency, medium frequency, and low frequency. And females were more likely to report emotional symptoms. In addition, high SSBs and takeaway consumption were associated with hyperactivity problems, emotional symptoms, conduct problems, peer problems, prosocial problems, and total difficulties. Other findings are shown in Tables 2, 3.

**Table 2:** The gender difference of SDQ scores among the samples, *n* (%).

**Variables**	**Total**	**Male**	**Female**	**χ^**2**^**
**Hyperactivity problems**				
Normal	23,139 (76.6)	12,621 (74.7)	10,518 (79.1)	89.34^**^
Borderline	3,109 (10.3)	1,830 (10.8)	1,279 (9.6)	
Abnormal	3,940 (13.1)	2,446 (14.5)	1,494 (11.2)	
**Emotional symptoms**				
Normal	21,499 (71.2)	12,698 (75.1)	8,801 (66.2)	325.21^**^
Borderline	3,103 (10.3)	1,632 (9.7)	1,471 (11.1)	
Abnormal	5,586 (18.5)	2,567 (15.2)	3,019 (22.7)	
**Conduct problems**				
Normal	25,663 (85.0)	14,026 (83.0)	11,637 (87.6)	130.25^**^
Borderline	2,439 (8.1)	1,496 (8.9)	943 (7.1)	
Abnormal	2,086 (6.9)	1,375 (8.1)	711 (5.3)	
**Peer problems**				
Normal	25,431 (84.2)	13,934 (82.5)	11,497 (86.5)	
Borderline	2,822 (9.3)	1,754 (10.4)	1,068 (8.0)	91.42^**^
Abnormal	1,935 (6.4)	1,209 (7.2)	726 (5.5)	
**Prosocial problems**				
Normal	22,030 (73.0)	11,836 (70.0)	10,194 (76.7)	
Borderline	4,929 (16.3)	2,936 (17.4)	1,993 (15.0)	197.71^**^
Abnormal	3,229 (10.7)	2,125 (12.6)	1,104 (8.3)	
**Total difficulties**				
Normal	24,216 (80.2)	13,599 (80.5)	10,617 (79.9)	
Borderline	3,327 (11.0)	1,869 (11.1)	1,458 (11.0)	4.46
Abnormal	2,645 (8.8)	1,429 (8.5)	1,216 (9.1)	

*SDQ, Strengths and Difficulties Questionnaire*.

*p < 0.05;

** *p < 0.01*.

**Table 3:** Scores on SDQ total difficulties and subscales, among sugar-sweetened beverages and takeaway.

	**Total difficulties**	**Emotional symptoms**	**Conduct problems**	**Hyperactivity problems**	**Peer problems**	**Prosocial problems**
**SSBs**						
High intake	19.40 ± 5.29	2.91 ± 2.33	2.38 ± 1.63	4.02 ± 2.11	3.15 ± 1.60	6.94 ± 2.11
Medium intake	18.30 ± 4.86	2.52 ± 2.18	1.99 ± 1.44	3.97 ± 2.19	2.88 ± 1.59	6.94 ± 2.06
Low intake	17.63 ± 4.89	2.16 ± 2.09	1.81 ± 1.42	3.98 ± 2.28	2.75 ± 1.64	6.93 ± 2.09
*p*-value	<0.01	<0.01	<0.01	<0.01	<0.01	0.038
**Takeaway**						
Never	18.06 ± 4.94	2.34 ± 2.14	1.94 ± 1.47	3.91 ± 2.23	2.89 ± 1.63	6.98 ± 2.08
Low frequency	18.57 ± 4.97	2.64 ± 2.23	2.07 ± 1.46	4.05 ± 2.14	2.90 ± 1.56	6.91 ± 2.04
Medium frequency	19.93 ± 5.34	3.24 ± 2.43	2.50 ± 1.65	4.33 ± 2.14	3.09 ± 1.65	6.76 ± 2.11
High frequency	20.48 ± 5.78	3.40 ± 2.59	2.80 ± 1.88	4.44 ± 2.18	3.36 ± 1.86	6.49 ± 2.49
*p*-value	<0.01	<0.01	<0.01	<0.01	<0.01	<0.01

*SDQ, Strengths and Difficulties Questionnaire; SSB, sugar-sweetened beverage*.

*p < 0.05; ^**^p < 0.01.

### The Relationship Between Sugar-Sweetened Beverages, Takeaway, and Psychological and Behavioral Problems

**Figure 1:**
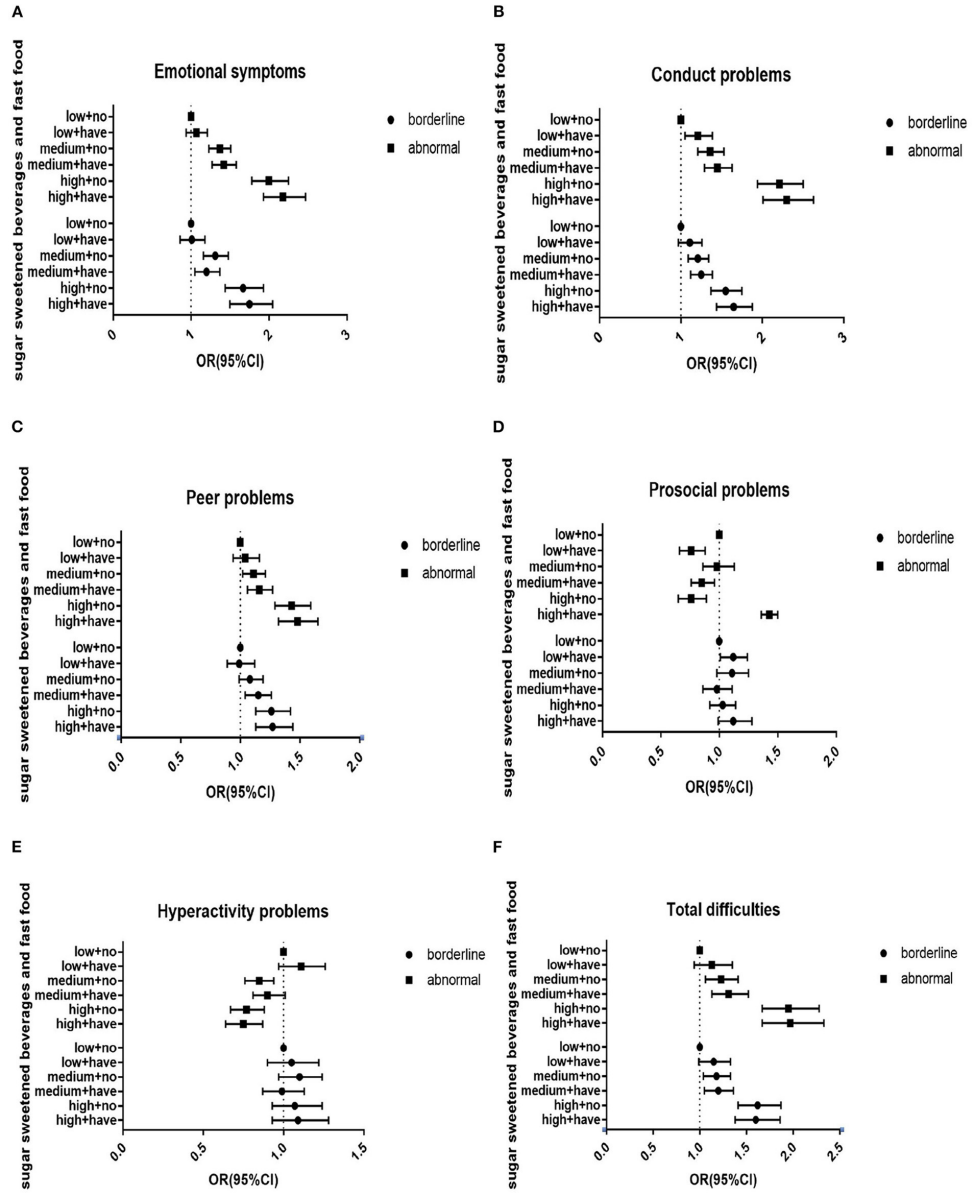
Interactive effects of sugar-sweetened beverages and takeaway pattern on psychological and behavioral problems among Chinese children and adolescents. **(A)** Interactive effects of sugar-sweetened beverages and takeaway pattern on emotional symptoms. **(B)** Interactive effects of sugar-sweetened beverages and takeaway pattern on conduct problems. **(C)** Interactive effects of sugar-sweetened beverages and takeaway pattern on peer problems. **(D)** Interactive effects of sugar-sweetened beverages and takeaway pattern on prosocial problems. **(E)** Interactive effects of sugar-sweetened beverages and takeaway pattern on hyperactivity problems. **(F)** Interactive effects of sugar-sweetened beverages and takeaway pattern on total difficulties. Low, low SSB intake; Medium, medium SSB intake; High, high SSB intake; Have, have takeaway consumption; No, no takeaway consumption. The model was controlled for age, gender, grade, residential areas, academic record, family economic level, and parents' education level. *p < 0.05; **p < 0.01.

In Table 4, after gender, grade, residential area, academic record, parents' educational level, and self-reported family economic level were adjusted for, more frequent intake of SSBs (OR = 2.23, 95%CI = 2.0–2.47, *p* < 0.01) and higher takeaway consumption (OR = 1.81, 95%CI = 1.66–1.97, *p* < 0.01) were associated with higher SDQ total difficulties scales. The same results about SSB consumption were also found in emotional symptoms (OR = 1.73, 95%CI = 1.60–1.87, *p* < 0.01), conduct problems (OR = 2.24, 95%CI = 2.05–2.44, *p* < 0.01), peer problems (OR = 1.67, 95%CI = 1.55–1.80, *p* < 0.01), and prosocial problems (OR = 1.11, 95%CI = 1.01–1.21, *p* < 0.01), except for hyperactivity problems (OR = 1.03, 95%CI = 0.93–1.14). In addition, higher takeaway consumption was also associated with higher SDQ scales. These results are shown in Table 4. Results from multivariate logistic regression analysis indicated that both takeaway dietary pattern and SSBs are independently associated with SDQ scores. Besides, they had a multiplied interaction impact between SSBs and takeaway dietary pattern on SDQ scores. Higher SSBs were more associated with total difficulties (OR = 3.10, 95%CI = 2.67–3.59, *p* < 0.01), emotional symptoms (OR = 2.10, 95%CI = 1.86–2.36, *p* < 0.01), conduct problems (OR = 3.24, 95%CI = 2.86–3.65, *p* < 0.01), peer problems (OR = 1.89, 95%CI = 1.69–2.12, *p* < 0.01), prosocial problems (OR = 1.26, 95%CI = 1.07–1.47, *p* < 0.01), hyperactivity problems (OR = 1.33, 95%CI = 1.15–1.55, *p* < 0.01), and higher takeaway dietary pattern than low and medium SSB consumption. These results are shown in Figure 1.

**Table 4:** Individual effects of sugar-sweetened beverages (SSBs) and takeaway patterns on psychological and behavioral problems.

	**SSBs (reference group: low SSB intake)**		**Takeaway pattern (reference group: never)**		
	**Medium**	**High**	**High**	**Medium**	**Low**
**Hyperactivity problems (reference group: normal)**					
Borderline	1.03 (0.94–1.12)	1.02 (0.94–1.10)	1.70 (1.30–2.23)^**^	1.35 (1.11–1.64)^*^	1.16 (1.07–1.25)^*^
Abnormal	1.27 (1.14–1.41)^**^	1.03 (0.93–1.14)	1.40 (1.07–1.83)^*^	1.51 (1.27–1.79)^**^	1.07 (0.99–1.15)
**Emotional symptoms (reference group: normal)**					
Borderline	1.20 (1.10–1.31)^**^	1.30 (1.21–1.42)^**^	1.69 (1.26–2.27)^**^	1.61 (1.33–1.95)^**^	1.23 (1.14–1.33)^**^
Abnormal	1.39 (1.25–1.54)^**^	1.73 (1.60–1.87)^**^	2.51 (2.03–3.10)^**^	2.26 (1.96–2.60)^**^	1.27 (1.19–1.35)^**^
**Conduct problems (reference group: normal)**					
Borderline	1.28 (1.15–1.42)^**^	1.73 (1.53–1.96)^**^	2.17 (1.64–2.86)^**^	1.86 (1.54–2.25)^**^	1.14 (1.04–1.24)^**^
Abnormal	1.25 (1.10–1.41)^**^	2.31 (2.02–2.64)^**^	2.82 (2.17–3.66)^**^	2.14 (1.77–2.58)	1.04 (0.94–1.14)
**Peer problems (reference group: normal)**					
Borderline	1.02 (0.92–1.12)^**^	1.15 (1.09–1.22)^**^	1.65 (1.25–2.17)^**^	1.34 (1.11–1.62)^**^	1.01 (0.93–1.09)
Abnormal	0.98 (0.87–1.09)	1.27 (1.14–1.43)^**^	1.84 (1.37–2.47)^**^	1.04 (0.83–1.32)	0.80 (0.72–0.88)^**^
**Prosocial problems (reference group: normal)**					
Borderline	0.92 (0.84–1.0)	1.12 (1.04–1.20)^**^	1.31 (1.02–1.68)^*^	1.31 (1.53–2.14)^**^	1.05 (0.97–1.13)
Abnormal	1.02 (0.92–1.14)	1.11 (1.01–1.21)^*^	2.34 (1.81–3.03)^**^	1.88 (1.56–2.26)^**^	1.16 (1.06–1.26)^**^
**Total difficulties (reference group: normal)**					
Borderline	1.15 (1.06–1.26)^**^	1.59 (1.44–1.75)^**^	1.53 (1.17–2.01)^**^	1.80 (1.53–2.14)^**^	1.05 (0.97–1.13)
Abnormal	1.38 (1.26–1.52)^**^	2.23 (2.00–2.47)^**^	2.34 (1.81–3.03)^**^	1.88 (1.56–2.26)^**^	1.16 (1.06–1.26)^**^

*The model was controlled for age, gender, grade, residential areas, academic record, family economic level, and parents' education level*.

* *p < 0.05*;

** *p < 0.01*.

## Discussion

Our study demonstrated the correlations between SSBs, takeaway dietary pattern, and PBPs in Chinese children and adolescents. After gender, age, family economic level, parents' education level and academic record, and residential area were adjusted for, SSBs and takeaway eating patterns were found to have individual and interactive effects on PBPs. In consideration of the discrepancies in unhealthy eating patterns for emotional and behavioral problems between Asians and other ethnicities, the results of this study may help us explore the influence of SSBs and takeaways on psychological behavioral problems in eastern dietary patterns.

Demographic characteristics and conditions known or considered to be correlates of psychological behavioral problems were measured; the consumption of SSBs, takeaways consumption, and psychological behavioral problems both associated with a variety of sociodemographic characteristics, which may confound the essential association between SSBs, takeaway consumption, and psychological behavioral problems (45). Children and adolescents with lower economic levels were at risk for poor diet status, for example, lower intake of fruits and vegetables, and higher intake of unhealthy snacks, fast food, and SSBs. A possible explanation was that the relative low prices on SSBs could explain why lower economic levels were associated with higher SSB intake, and lower economic levels were not enough for healthy dietary. Others include gender, parents' educational levels, age, and grade. This might imply that adolescents' personal economic levels should be included in the studies of health-related behaviors. So we determined the selection of covariates by referring to previous studies and literatures, as well as the preliminary experimental results of the research group (37). In addition, gender and age effects differ in behavioral and emotional problems. Gender differences were found in the distribution of PBPs. Compared with girls, boys were more likely to score higher conduct problems, peer problems, hyperactivity problems, prosocial problems, and total difficulties. These results were similar to previous studies (47–49). In terms of emotional problems, girls are more likely to have higher score than boys (47, 48). Similarly, children and adolescents with low family income, compared with high family income, were more likely to report PBPs; and those with low parental education were also more likely to have PBPs than those with high parental education. Our study revealed that SSB consumption and takeaway dietary pattern brought an individual and interactive relationship risk of PBPs after adjustments for confounders.

SSBs have been found to be associated with a higher prevalence of mental health problems (15, 19, 32). The most consumed non-alcoholic beverages were SSBs, coffee, and tea and may have important health consequences; others such as energy drinks also have same results (50). Schwartz et al. conducted a survey of 1,649 U.S. children about the Health Behavior Survey and Hyperactivity Disorder questionnaire and found that higher SSB intake was associated with an increased risk of ADHD (22). This is similar to Alsamghan's result: a significance association was found with risk of hyperactivity/inattention who consumed energy drinks (51). More important, some of the bad behaviors established in childhood, such as the SSB eating pattern, may persist into adulthood (52, 53). Considering that students spend most of their time in school, the type of food sold or served in schools is an important environmental factor affecting children's eating patterns, and governments should restrict SSB provision in schools to promote healthy eating behavior among young people (54), so it is important for school leaders to take action to prevent adolescents' SSBs and takeaway dietary consumption. The results should catch the attention from not only parents and policymakers but also the producers and sellers of SSBs. In our study, we have not found a relationship between SSB consumption and hyperactivity problems. One possible reason was that our SSB scores included numerous varieties; some of these beverages were positively correlated with hyperactivity and prosocial problems, while others were negatively correlated, so there might be an offsetting effect.

The PBP consequences of SSBs can be summarized as follows: a) sugar intolerance (physical discomfort after eating or drinking sugary foods); b) body's reactive hypoglycemia after ingestion; and c) decrease in intake of essential micronutrients. We extracted some of the more important results: if psychological problems are not paid attention to, these can easily lead to depression and mental disorders. So how are sugary drinks related to depression and mental disorders? Some researches have verified their correlated mechanisms. Continual consumption of SSBs, especially diet SSBs, may increase the risk of depression, while coffee consumption may reduce the risk. We hypothesized that there are several possible mechanisms linking sugar intake and anxiety/depression, including oxidative stress response (55) and serotonin (5-HT) mechanisms (56). Other researches have discussed the connection between high sugary behavior and mental health, including high-sensitivity C-reactive protein (hsCRP) (57, 58). Other studies demonstrated that the consumption of sodium benzoate (found in beverages) impairing memory and motor coordination, reducing glutathione, increasing the malondialdehyde level in the brain, and inducing ADHD in children is emphasized (59). In Yu's study, compared with those who did not consume SSBs, children who consumed SSBs at moderate levels and high levels were associated with having ADHD (60); and another study conducted by Howard revealed that an “unhealthy,” western-style preference diet (i.e., more meat and sweets and fewer vegetables and fruits) was associated with ADHD (61).

The theory of planned behavior (TPB) states that SSB intake causes not only PBPs but also a range of behaviors (62) and that they influence one other (31, 63). Experimental results showed that more frequent intake of SSBs was associated with higher prevalence of middle and lower annual household income, lower frequency of physical activity, more takeaway dietary behavior, more fast-food behavior, and more frequency of screen time. And the results from the human sample show the same result of sensitivity to reward and adolescents' unhealthy snacking and drinking behaviors (64). So we also proposed the takeaway dietary pattern and found that takeaway dietary pattern was correlated with PBPs. Our results further suggest that psycho-pathological symptoms, including emotional, conduct and prosocial problems, were significantly associated with SSB consumption and takeaway dietary pattern in a dose-dependent manner. Specifically, after variables were controlled for, takeaway and SSB eating patterns are associated with increased risk of psycho-pathological symptoms; these results were consistent with previous cross-sectional studies (29, 65). One possible reason is that people find healthier foods to be tastier and more popular than unhealthy foods (66).

We found an obvious interaction between high SSBs and takeaway dietary pattern on PBPs. High SSB intake causes an increase in the risk of PBPs in students with high takeaway dietary pattern compared with students with low takeaway dietary pattern. The possible underlying mechanisms for this interaction are complex. One possible reason was de Bruijn's research: TPB also means that health behaviors in youth tend to cluster and that interventions that succeed in inducing positive changes in cognition and intention in a behavior may lead to positive changes in an aggregation behavior (62). Another possible reason was a positive correlation between SSBs and takeaway diet pattern (67). Higher takeaway dietary pattern was associated with higher SSB consumption (63). The unhealthy association is thought to be caused by high exposure to food and drink advertisements during screen time. Unhealthy home food availability increased takeaway diet pattern, further influencing the consumption of SSBs (68). Because when some take the takeaway diet pattern, they do not notice what they were eating, which could eventually lead to overconsumption (69). So we could think of the interactive correlation between SSBs and UDP on PBPs (37).

Our study has several limitations. First, this study is cross-sectional research; it cannot detect a causal relationship and could not judge causality or direction, and a future longitudinal study is suggested. Second, SSBs and takeaway consumption were acquired through self-reporting, which may have caused recall bias. Third, there were many factors influencing PBPs, and we only have explored two of them, so further researches will pay attention to other factors. Despite these limitations, our research has some strengths. First, most importantly, this is a survey of Chinese children and adolescents, and the results of our study can be applied to public health and clinical practice in other populations. The limitations of other reports were the small sample sizes. Our research aims to explore the mental health behavioral problems of children and adolescents through a large sample and multi-age perspectives. In addition, 30,188 adolescents were sampled from 14 schools in Shenzhen, China, with a wide sampling range and a large sample size. The cluster stratified random sampling method was used to identify the sample, and primary schools, as well as middle schools, were included in this multilevel survey. These data were somewhat representative. We also analyzed several potential confounders. In this study, we analyzed the interactive effect to explore the correlation between SSBs, takeaway consumption, and PBPs, further demonstrating that we should pay close attention to the factors influencing children's and adolescents' mental health. Our study can also provide a good theoretical basis for the follow-up large sample of children and adolescents regarding SSBs, takeaway food, and mental health issues.

In summary, this study offers discernment into the association between SSB consumption, takeaway consumption, and PBPs among Chinese children and adolescents. Our results suggested that higher SSB consumption and higher takeaway consumption were all positively associated with PBPs. Also, the interactive relationship between SSBs and takeaway consumption was stronger than SSB consumption and takeaway consumption individually. This is a public health issue that cannot be ignored, given China's large population base and the growing trend of SSB consumption and takeaway consumption.

## Data Availability Statement

The datasets generated for this study are available on request to the corresponding author.

## Ethics Statement

The studies involving human participants were reviewed and approved by the Ethics Committee of Anhui Medical University. Written informed consent to participate in this study was provided by the participants' legal guardian/next of kin.

## Author Contributions

FT designed the study. YZ, XW, ST, QW, RW, TL, and QZ performed the survey research. YZ, XW, ST, and QW analyzed the data. YZ drafted the manuscript. All authors read and approval the final manuscript.

## Conflict of Interest

The authors declare that the research was conducted in the absence of any commercial or financial relationships that could be construed as a potential conflict of interest.

## Publisher's Note

All claims expressed in this article are solely those of the authors and do not necessarily represent those of their affiliated organizations, or those of the publisher, the editors and the reviewers. Any product that may be evaluated in this article, or claim that may be made by its manufacturer, is not guaranteed or endorsed by the publisher.

## References

[1] Hu FB Malik VS. Sugar-sweetened beverages and risk of obesity and type 2 diabetes: epidemiologic evidence. Physiol Behav. (2010) 100:47–54. 10.1016/j.physbeh.2010.01.036 20138901 PMC2862460

[2] Yang Q Zhang Z Gregg EW Flanders WD Merritt R Hu FB. Added sugar intake and cardiovascular diseases mortality among U.S. adults. JAMA Intern Med. (2014) 174:516–24. 10.1001/jamainternmed.2013.13563 24493081 PMC10910551

[3] Ebbeling CB Feldman HA Chomitz VR Antonelli TA Gortmaker SL Osganian SK. A randomized trial of sugar-sweetened beverages and adolescent body weight. N Engl J Med. (2012) 367:1407–16. 10.1056/NEJMoa1203388 22998339 PMC3494993

[4] Paglia L Friuli S Colombo S Paglia M. The effect of added sugars on children's health outcomes: obesity, obstructive sleep apnea syndrome (OSAS), attention-deficit/ hyperactivity disorder (ADHD) and Chronic Diseases. Eur J Pediatr Dent. (2019) 20:127–32. 10.23804/ejpd.2019.20.02.09 31246089

[5] Ruff RR. Sugar-sweetened beverage consumption is linked to global adult morbidity and mortality through diabetes mellitus, cardiovascular disease and adiposity-related cancers. Evid Based Med. (2015) 20:223–4. 10.1136/ebmed-2015-110267 26442567

[6] Malik VS Hu FB. Sugar-sweetened beverages and cardio-metabolic health: an update of the evidence. Nutrients. (2019) 11:1840. 10.3390/nu11081840 31398911 PMC6723421

[7] Arnold LE Lofthouse N Hurt E. Artificial food colors and attention-deficit/hyperactivity symptoms: conclusions to dye for. Neurotherapeutics. (2012) 9:599–609. 10.1007/s13311-012-0133-x 22864801 PMC3441937

[8] Woo HD Kim DW Hong YS Kim YM Seo JH Choe BM. Dietary patterns in children with attention deficit/hyperactivity disorder (ADHD). Nutrients. (2014) 6:1539–53. 10.3390/nu6041539 24736898 PMC4011050

[9] Malik VS Pan A Willett WC Hu FB. Sugar-sweetened beverages and weight gain in children and adults: a systematic review and meta-analysis. Am J Clin Nutr. (2013) 98:1084–102. 10.3945/ajcn.113.058362 23966427 PMC3778861

[10] Hardy LL Bell J Bauman A Mihrshahi S. Association between adolescents' consumption of total and different types of sugar-sweetened beverages with oral health impacts and weight status. Aust N Z J Public Health. (2018) 42:22–6. 10.1111/1753-6405.12749 29165908

[11] Mesirow MS Welsh JA. Changing beverage consumption patterns have resulted in fewer liquid calories in the diets of US children: National Health and Nutrition Examination Survey 2001-2010. J Acad Nutr Diet. (2015) 115:559–66. 10.1016/j.jand.2014.09.004 25441966

[12] Park S Blanck HM Sherry B Brener N O'Toole T. Factors associated with sugar-sweetened beverage intake among United States high school students. J. Nutr. (2012) 142:306–12. 10.3945/jn.111.148536 22223568 PMC4532336

[13] Knüppel A Shipley MJ Llewellyn CH Brunner EJ. Sugar intake from sweet food and beverages, common mental disorder and depression: prospective findings from the Whitehall II study. Sci Rep. (2017) 7:6287. 10.1038/s41598-017-05649-7 28751637 PMC5532289

[14] DeSalvo KB Olson R Casavale KO. Dietary guidelines for Americans. JAMA. (2016) 315:457–8. 10.1001/jama.2015.18396 26746707

[15] Kadel P Schneider S Mata J. Soft drink consumption and mental health problems: longitudinal relations in children and adolescents. Soc Sci Med. (2020) 258:113123. 10.1016/j.socscimed.2020.113123 32593956

[16] Millichap JG Yee MM. The diet factor in attention-defificit/hyperactivity disorder. Pediatrics. (2012) 129:330–7. 10.1542/peds.2011-2199 22232312

[17] Zhang X Huang X Xiao Y Jing D Huang Y Chen L. Daily intake of soft drinks is associated with symptoms of anxiety and depression in Chinese adolescents. Public Health Nutr. (2019) 22:2553–60. 10.1017/S1368980019001009 31097051 PMC10260691

[18] Pérez-Ara MÁ Gili M Visser M Penninx BWJH Brouwer IA Watkins E. Associations of non-alcoholic beverages with major depressive disorder history and depressive symptoms clusters in a sample of overweight adults. Nutrients. (2020) 12:3202. 10.3390/nu12103202 33092067 PMC7589496

[19] Freije SL Senter CC Avery AD Hawes SE Jones-Smith JC. Association between consumption of sugar-sweetened beverages and 100% fruit juice with poor mental health among US adults in 11 US States and the District of Columbia. Prev Chronic Dis. (2021) 18:E51. 10.5888/pcd18.200574 34014815 PMC8139445

[20] Lin PY Lin FY Chen TC Chen WL Doong JY Shikanai S. Relationship between sugar intake and obesity among school-age children in Kaohsiung, Taiwan. J Nutr Sci Vitaminol. (2016) 62:310–16. 10.3177/jnsv.62.310 27928117

[21] Campos V Despland C Brandejsky V Kreis R Schneiter P Chiolero A. Sugar-and artificially sweetened beverages and intrahepatic fat: a randomized controlled trial. Obesity. (2015) 23:2335–9. 10.1002/oby.21310 26727115

[22] Schwartz DL Gilstad-Hayden K Carroll-Scott A Grilo SA McCaslin C Schwartz M. Energy drinks and youth self-reported hyperactivity/inattention symptoms. Acad. Pediatr. (2015) 15:297–304. 10.1016/j.acap.2014.11.006 25676784 PMC4772143

[23] Zeng Q Zeng Y. Eating out and getting fat? A comparative study between urban and rural China. Appetite. (2018) 120:409–15. 10.1016/j.appet.2017.09.027 28964905

[24] Wang H Yu Y Tian X. Does eating-away-from-home increase the risk of a metabolic syndrome diagnosis?. Int J Environ Res Public Health. (2019) 16:575. 10.3390/ijerph16040575 30781483 PMC6406498

[25] Wang Z Zhai F Du S Popkin B. Dynamic shifts in Chinese eating behaviors. Asia Pac. J. Clin. Nutr. (2008) 17:123–130. 10.1096/fasebj.22.1_supplement.678.4 18364337

[26] Zhai FY Du SF Wang ZH Zhang JG Du WW Popkin BM. Dynamics of the Chinese diet and the role of urbanicity, 1991-2011. Obes Rev. (2014) 15(Suppl. 1):16–26. 10.1111/obr.12124 24341755 PMC3868998

[27] Pereira MA Kartashov AI Ebbeling CB Van Horn L Slattery ML Jacobs DR. Fast-food habits, weight gain, and insulin resistance (the CARDIA study): 15-year prospective analysis. Lancet. (2005) 365:36–42. 10.1016/S0140-6736(04)17663-0 15639678

[28] Lee J Allen J. Gender differences in healthy and unhealthy food consumption and its relationship with depression in young adulthood. Community Ment Health J. (2021) 57:898–909. 10.1007/s10597-020-00672-x 32602082

[29] Ren J Luo X Zhao X Yang W Yang M Wang Y. Takeaway food in Chengdu, Sichuan province, China: composition and nutritional value. Asia Pac J Clin Nutr. (2020) 29:883–98. 10.6133/apjcn.202012_29(4).0025 33377384

[30] Okuyama K Li X Abe T Hamano T Franks PW Nabika T. Fast food outlets, physical activity facilities, and obesity among adults: a nationwide longitudinal study from Sweden. Int J Obes. (2020) 44:1703–11. 10.1038/s41366-020-0588-5 32424265

[31] Azadbakht L Esmaillzadeh A. Dietary patterns and attention deficit hyperactivity disorder among Iranian children. Nutrition. (2012) 28:242–9. 10.1016/j.nut.2011.05.018 21868196

[32] Burrows T Hides L Brown R Dayas CV Kay-Lambkin F. Differences in dietary preferences, personality and mental health in Australian adults with and without food addiction. Nutrients. (2017) 9:285. 10.3390/nu9030285 28294965 PMC5372948

[33] Kulkarni A Swinburn B Utter J. Associations between diet quality and mental health in socially disadvantaged New Zealand adolescents. Eur J Clin Nutr. (2015) 69:79–83. 10.1038/ejcn.2014.130 25028085

[34] Ríos-Hernández A Alda JA Farran-Codina A Ferreira-García E Izquierdo-Pulido M. The mediterranean diet and ADHD in children and adolescents. Pediatrics. (2017) 139:e20162027. 10.1542/peds.2016-2027 28138007

[35] Kim KM Lim MH Kwon HJ Yoo SJ Kim EJ Kim JW. Associations between attention-deficit/hyperactivity disorder symptoms and dietary habits in elementary school children. Appetite. (2018) 127:274–79. 10.1016/j.appet.2018.05.004 29758272

[36] O'Neil A Quirk SE Housden S Brennan SL Williams LJ Pasco JA. Relationship between diet and mental health in children and adolescents: a systematic review. Am J Public Health. (2014) 104:e31–42. 10.2105/AJPH.2014.302110 25208008 PMC4167107

[37] Xu HL Sun Y Wan YH Zhang SC Xu HQ Yang R. Eating pattern and psychological symptoms: a cross-sectional study based on a national large sample of Chinese adolescents. J Affect Disord. (2018) 244:155–63. 10.1016/j.jad.2018.10.090 30340102

[38] Foo LH Lee YH Suhaida CY Hills AP. Correlates of sugar-sweetened beverage consumption of Malaysian preschoolers aged 3 to 6 years. BMC Public Health. (2020) 20:552. 10.1186/s12889-020-08461-7 32334561 PMC7183579

[39] Shareck M Lewis D Smith NR Clary C Cummins S. Associations between home and school neighbourhood food environments and adolescents' fast-food and sugar-sweetened beverage intakes: findings from the Olympic Regeneration in East London (ORiEL) Study. Public Health Nutr. (2018) 21:2842–51. 10.1017/S1368980018001477 29962364 PMC10260913

[40] Skidmore P Welch A van Sluijs E Jones A Harvey I Harrison F. Impact of neighbourhood food environment on food consumption in children aged 9-10 years in the UK SPEEDY (Sport, Physical Activity and Eating behaviour: Environmental Determinants in Young people) study. Public Health Nutr. (2010) 13:1022–30. 10.1017/S1368980009992035 20082745 PMC3164802

[41] Goodman R. The strengths and difficulties questionnaire: a research note. J Child Psychol Psychiatry. (1997) 38:581–6. 10.1111/j.1469-7610.1997.tb01545.x 9255702

[42] Matsuishi T Nagano M Araki Y Tanaka Y Iwasaki M Yamashita Y. Scale properties of the Japanese version of the Strengths and Difficulties Questionnaire (SDQ): a study of infant and school children in community samples. Brain Dev. (2008) 30:410–5. 10.1016/j.braindev.2007.12.003 18226867

[43] Goodman R. Psychometric properties of the strengths and difficulties questionnaire. J Am Acad Child Adolesc Psychiatry. (2001) 40:1337–45. 10.1097/00004583-200111000-00015 11699809

[44] Gao X Shi W Zhai Y He L Shi X. Results of the parent-rated strengths and difficulties questionnaire in 22,108 primary school students from 8 provinces of China. Shanghai Arch Psychiatry. (2013) 25:364–74. 10.3969/j.issn.1002-0829.2013.06.005 24991179 PMC4054577

[45] Geng M Jiang L Wu X Ding P Liu W Liu M. Sugar-sweetened beverages consumption are associated with behavioral problems among preschoolers: a population based cross-sectional study in China. J Affect Disord. (2020) 265:519–25. 10.1016/j.jad.2020.01.076 32090780

[46] Andrew AB Peggy A Robert S. Relationship between insulin resistance-associated metabolic parameters and anthropometric measurements with sugar-sweetened beverage intake and physical activity levels in US adolescents findings from the 1999-2004 National Health and Nutrition Examination Survey. Arch Pediatr Adolesc Med. (2009) 163:328–35. 10.1001/archpediatrics.2009.21 19349561 PMC4264593

[47] Bibou-Nakou I Markos A Padeliadu S Chatzilampou P Ververidou S. Multi-informant evaluation of students' psychosocial status through SDQ in a national Greek sample. Child Youth Serv Rev. (2019) 96:47–54. 10.1016/j.childyouth.2018.11.022

[48] Idris IB Barlow J Dolan A. A longitudinal study of emotional and behavioral problems among Malaysian school children. Ann Glob Health. (2019) 85:30. 10.5334/aogh.2336 30873768 PMC6634446

[49] Shibata Y Okada K Fukumoto R Nomura K. Psychometric properties of the parent and teacher forms of the Japanese version of the strengths and difficulties questionnaire. Brain Dev. (2015) 37:501–7. 10.1016/j.braindev.2014.08.001 25172302

[50] Al-Shaar L Vercammen K Lu C Richardson S Tamez M Mattei J. Health effects and public health concerns of energy drink consumption in the United States: a mini-review. Front Public Health. (2017) 5:225. 10.3389/fpubh.2017.00225 28913331 PMC5583516

[51] Alsamghan AS Bharti RK Alshbqe AAM AlahmariM S Alqahtani ASM Ayidh FAN. Energy drinks consumption and its relationship with hyperactivity/inattention behaviour among the intermediate and high school male and female students. J. Evid. Based Med. Healthc. (2016) 3:4081–6. 10.18410/jebmh/2016/872

[52] Appannah G Murray K Trapp G Dymock M Oddy WH Ambrosini GL. Dietary pattern trajectories across adolescence and early adulthood and their associations with childhood and parental factors. Am J Clin Nutr. (2020) 113:36–46. 10.1093/ajcn/nqaa281 33181820

[53] Hur YI Park H Kang JH Lee HA Song HJ Lee HJ. Associations between sugar intake from different food sources and adiposity or cardio-metabolic risk in childhood and adolescence: the Korean child-adolescent cohort study. Nutrients. (2015) 8:20. 10.3390/nu8010020 26729156 PMC4728634

[54] von Philipsborn P Stratil JM Burns J Busert LK Pfadenhauer LM Polus S. Environmental interventions to reduce the consumption of sugar-sweetened beverages and their effects on health. Cochrane Database Syst Rev. (2019) 6:CD012292. 10.1002/14651858.CD012292.pub2 31194900 PMC6564085

[55] Maes M Kubera M Obuchowiczwa E Goehler L Brzeszcz J. Depression's multiple comorbidities explained by (neuro) inflammatory and oxidative & nitrosative stress pathways. Neuroendocrinol Lett. (2011) 32:7–24. 21407167

[56] Martins J Brijesh S. Phytochemistry and pharmacology of anti-depressant medicinal plants: a review. Biomed Pharmacother. (2018) 104:343–65. 10.1016/j.biopha.2018.05.044 29778018

[57] Mazidi M Kengne AP Mikhailidis DP Arrigo F Banach CM. Effects of selected dietary constituents on high sensitivity C-reactive protein levels in U.S. adults. Ann Med. (2018) 50:1–6. 10.1080/07853890.2017.1325967 28462631

[58] Aeberli I Gerber PA Hochuli M. Low to moderate sugar-sweetened beverage consumption impairs glucose and lipid metabolism and promotes inflammation in healthy young men: a randomized controlled trial. Am J Clin Nutr. (2011) 94:479–85. 10.3945/ajcn.111.013540 21677052

[59] Anjum I Jaffery SS Fayyaz M Wajid A Ans AH. Sugar beverages and dietary sodas impact on brain health: a mini literature review. Cureus. (2018) 10:e2756. 10.7759/cureus.2756 30094113 PMC6080735

[60] Yu CJ Du JC Chiou HC Feng CC Chung MY Yang W. Sugar-sweetened beverage consumption is adversely associated with childhood attention deficit/hyperactivity disorder. Int J Environ Res Public Health. (2016) 13:678. 10.3390/ijerph13070678 27384573 PMC4962219

[61] Howard AL Robinson M Smith GJ Ambrosini GL Piek JP Oddy WH. ADHD is associated with a “Western” dietary pattern in adolescents. J Atten Disord. (2011) 15:403–11. 10.1177/1087054710365990 20631199

[62] de Bruijn G van den Putte B. Adolescent soft drink consumption, television viewing and habit strength. Investigating clustering effects in the Theory of Planned Behaviour. Appetite. (2009) 53:66–75. 10.1016/j.appet.2009.05.008 19463873

[63] Larson N De-Wolfe J Story M Neumark-Sztainer D. Adolescent consumption of sports and energy drinks: linkages to higher physical activity, unhealthy beverage patterns, cigarette smoking, and screen media use. J Nutr Educ Behav. (2014) 46:181–7. 10.1016/j.jneb.2014.02.008 24809865 PMC4023868

[64] De Cock N Van Lippevelde W Goossens L De Clercq B Vangeel J Lachat C. Sensitivity to reward and adolescents' unhealthy snacking and drinking behavior: the role of hedonic eating styles and availability. Int J Behav Nutr Phys Act. (2016) 13:17. 10.1186/s12966-016-0341-6 26861539 PMC4748632

[65] Sinclair R Millar L Allender S Snowdon W Waqa G Jacka F. The cross-sectional association between diet quality and depressive symptomology amongst Fijian adolescents. PLoS ONE. (2016) 11:e0161709. 10.1371/journal.pone.0161709 27560960 PMC4999057

[66] Werle COC Trendel O Ardito G. Unhealthy food is not tastier for everybody: the ‘healthy=tasty' French intuition. Food Qual Prefer. (2013) 28:116–21. 10.1016/j.foodqual.2012.07.007

[67] Gan WY Mohamed SF Law LS. Unhealthy lifestyle associated with higher intake of sugar-sweetened beverages among Malaysian school-aged adolescents. Int J Environ Res Public Health. (2019) 16:2785. 10.3390/ijerph16152785 31382672 PMC6696103

[68] Garber AK Lustig RH. Is fast food addictive? Curr Drug Abuse Rev. (2011) 4:146–62. 10.2174/1874473711104030146 21999689

[69] Wang H Zhong J Hu R Fiona B Yu M Du H. Prevalence of high screen time and associated factors among students: a cross-sectional study in Zhejiang, China. BMJ Open. (2018) 8:e021493. 10.1136/bmjopen-2018-021493 29921687 PMC6009552

